# People with multimorbidity in outpatient care: patient-focused and needs-oriented healthcare management (MamBo) – protocol for a multiperspective evaluation study

**DOI:** 10.1186/s12913-020-05178-6

**Published:** 2020-04-13

**Authors:** Simone Richter, Ibrahim Demirer, Kyung-Eun Choi, Johannes Hartrampf, Holger Pfaff, Ute Karbach

**Affiliations:** 1 Institute of Medical Sociology, Health Services Research, and Rehabilitation Science (IMVR), Faculty of Human Sciences and Faculty of Medicine, University of Cologne, Eupener Strasse 129, 50933 Cologne, Germany; 2 Professional association of ophthalmologists in Germany e.V, Düsseldorf, Germany; 3 Faculty of Rehabilitation Sciences, TU Dortmund University, Dortmund, Germany

**Keywords:** Multimorbid patients, Primary care, Outpatient care, Care management, House calls

## Abstract

**Background:**

With demographic change, the number of noncommunicable diseases, chronic diseases and multimorbidity is increasing, and so is the demand for health services. This represents a further challenge for the healthcare system. An adequate and efficient treatment of multimorbid patients requires a well-structured, informed and cross-indicated treatment. Therefore, a new form of coordinated, managed and cross-sectoral care for multimorbid patients - the “MamBo” care model - has been developed. Along with the implementation of MamBo, a process and outcome evaluation will be carried out, which is described in this study protocol. The aim of the study is to evaluate the care model according to its implementation process and effectiveness.

**Methods:**

The MamBo-care model will be evaluated in multi-perspective terms. Thus, a process and outcome evaluation with several data sources will be conducted: (1) Annual focus groups and individual interviews with those involved in the process. (2) Various primary data, including surveys of patients, physicians and practice staff at the time of enrolment and 1 year later to enable pre-post comparison. (3) Claim data from the health insurance of the MamBo population in comparison to a comparative population, formed by the propensity score matching method. (4) Process data of the care management. The analysis of qualitative data will be carried out by content analysis according to Mayring. For the analysis of the quantitative data, multivariate analyses are planned.

**Discussion:**

A new form of coordinated care has been introduced to improve intersectoral care of multimorbid patients and reduce the workload on physicians. The effects of the MamBo care model are being investigated for patients, physicians and the cost units. The results will form the basis for the decision whether the care model should be transferred to standard care and what needs to be taken into account for implementation.

**Trial registration:**

The study was retrospectively registered in the German Register for Clinical Studies (DRKS00014047) on June 28, 2019.

## Background

In the context of demographic change, one of the most important tasks in the future will probably be to guarantee an adequate care for chronically ill and multimorbid patients [1]. As a growing life expectancy is accompanied by increasing life years and multimorbidity, the ageing society is likely to pose further challenges to the health care system [2]. In Germany, the number of people aged 65 and over is rising constantly while every second person at the age of 65 suffers at least from one chronic illness [3]. Multimorbidity is associated with an increased contact with doctors, more frequent and longer hospital stays and an increased number of drug prescriptions (polypharmacy) [4, 5].

The health care of chronically ill and multimorbid patients is very complex, as several physicians and further health as well as social professionals need to be involved in their care. The integration of general medical and specialist care, outpatient, inpatient and nursing care poses a challenge for the health care but in particular for the general practitioners (GP). They are entrusted with the coordination and, thus, they must always be well informed and communicate with all the professionals involved [1]. Adequate and efficient treatment, therefore, requires well -structured, −coordinated and -informed care. However, the highly complex health care system in Germany makes coordinated action more difficult due to its strong segmentation into the outpatient, hospital and nursing care sectors. In addition, there is no overall legal regulation in Germany for gatekeeper strategies. The given structural conditions of primary and secondary care are accompanied by interface problems between the care sectors and between occupational groups in the outpatient sector [6]. The advantages of structured care are proven by the experiences with the Disease Management Programmes (DMP) in Germany [7]. However, DMPs only address isolated disease. There are currently no structures that address the needs of multimorbid patients.

With the Innovation Fund of the Federal Joint Committee of Germany, new models of care that go beyond the regular care are promoted. Every funded study is evaluated under everyday conditions [8]. The findings of the evaluation serve the Federal Joint Committee and the national legislator as a basis for decision-making. The aim is to transfer successful care models to standard health care.

**Fig. 1:**
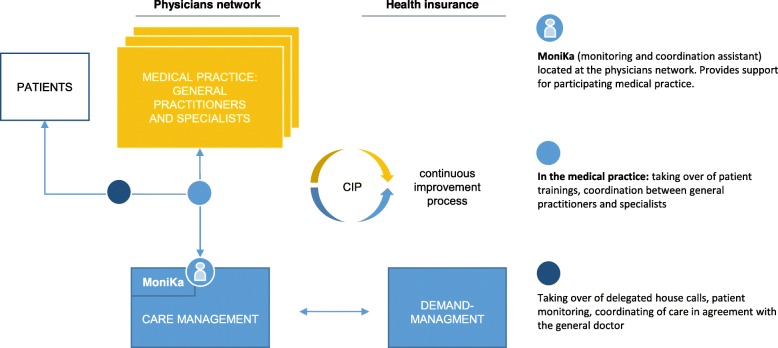
MamBo-structure

Building upon this, a new form of coordinated, cross-sectoral care for multimorbid patients was developed which is currently being implemented and evaluated. The project supported by the G-BA Innovation Fund is titled: “People with Multimorbidity in Outpatient Care: Patient-Focused and Needs-Oriented Healthcare Management (MamBo)”. MamBo consists of a care management (CM), including responsible persons for the management and up to five monitoring and coordination assistants (MoniKa) set up in a collaborating Regional Health Network (RGL) and a demand management (DM) established on the part of collaborating health insurance. The MoniKas are working on a cross-practice basis and taking over patient-oriented and coordinative tasks (e.g. house calls, patient training, coordination tasks) that can be delegated by doctors. In a continuous improvement process (CIP), the CM and DM, together with the medical practices involved, develop solutions for collective and patients oriented needs (see Fig. 1). An external organisational consulting company supports the initiators of the care model in the implementation.

The overarching objective of this study is to evaluate the care model MamBo in multi-perspective terms according to its implementation process and effectiveness. This raises the question of which factors seem to be beneficial or inhibiting for the implementation of the innovation and whether the quality and efficiency of care in the region have changed over time. The results will be used in political decision-making to assess whether the model for its transfer to general health care is sufficient, appropriate, economic and necessary.

## Methods/design

### Setting

The new care model MamBo is introduced in a physician’s network in the region of Leverkusen, a small metropolis in North Rhine-Westphalia (Germany). The implementation takes place in established general practitioners or specialist practices. Only physicians who are part of the physicians’ network can participate in MamBo. Currently, it is estimated that approx. 40 physicians and approx. 160 practice staff will participate in the care model. Inturn, approx. 2615 multimorbid patients with at least three chronic diseases and insured with PronovaBKK will be recruited by the participating physicians for the MamBo program. Patients in oncological or palliative treatment are excluded. The initial period of the MamBo-project was set for 3 years (07.2017–06.2020). After applying for an extension of 9 months, due to delays at the start of the study and initial recruitment difficulties, the study now ends on 31 March 2021.

The presented study is a multiperspective evaluation study including a qualitative and a quantitative evaluation design. Qualitative data for process evaluation and quantitative data for outcome evaluation are collected simultaneously. Different domains of the quantitative and qualitative evaluation design are listed in Table 1.

**Table 1:** Different domains of the multiperspective evaluation study

Evaluation type	Data source		time of data collection
Process evaluation	focus groups	participating physicians	annual, during the intervention phase
		monitoring and coordination assistants	
	semi- structured interviews	demand management	
		care management	
		management consultancy	once after the implementation
Outcome evaluation	primary data	questionnaire of participating physicians (n ~ 40)	two time-points during the intervention phase
		questionnaire of participating practice staff (n ~ 160)	
		questionnaire of participating patients (t0: n ~ 2460; t0 & t1: n ~ 1500)	
		questionnaire of non-participating physicians within the physician’s network (n ~ 60)	one time-point during the intervention phase
	secondary data	statutory health insurance claim data (group 1: n ~ 1.975; group 2: n ~ 2524)	during and at the end of the intervention phase
		RGL- process data	

### Qualitative evaluation

#### Qualitative evaluation design

Annual focus groups with participating physicians will be conducted to identify factors influencing the implementation and adoption of the innovation. Additionally, the operative actors will be interviewed. In this context focus groups with all employed MoniKas are held each year to gain deep insights into the role of the MoniKas and the complexity of the delegation process. Along with them, annual face-to-face interviews with the DM and the CM will be conducted to discuss the challenges associated with the implementation on a conceptual and organisational level. In addition, one-time expert interviews with representatives of the management consultancy provide deep insights into the complexity, challenges and benefits of change management as an implementation strategy in health care.

#### Sampling

The recruitment of all respondents is conducted purposely [9]. The selection of physicians for the focus groups is based on the Theory Rogers “Diffusion of innovation” [10]. Focus groups with physicians that are active from the beginning (“early adopters”, within the first 6 months) are conducted separately from physicians that become active late (“late adopters”). The board of the RGL provides access to physicians relevant for the respective focus groups. Comprehensively, the research team sends out invitations, information about the procedure and the letters of consent to all participants of face-to-face interviews and focus groups via fax or e-mail.

#### Data collection and analysis

The focus groups and interviews will follow a semi-structured guideline to allow comparisons. For each main topic, open questions are designed. The main topics followed are based on questions regarding inhibiting & facilitating factors of the implementation, expected and perceived advantages, communication and cooperations, work organisation as well as the usefulness of change management for implementation. The guideline will be adopted according to the background and function of the interviewee.

According to process evaluation standards, the interviews are carried out in waves at different times of the projects’ progress. Thus, three focus groups (4–8 physicians each) with participating physicians are planned. One was carried out in June 2018 already and one in January 2019. The second focus group has been supplemented by three individual interviews with doctors in order to gain a deeper insight into the complexity of the implementation and to reach more doctors to share their opinions. If less than four physicians can be recruited for a joint appointment, but other physicians have expressed their interests, additional individual interviews will also be conducted in further data collection waves. The first focus group with MoniKas was carried out in summer 2019, as only at this time an appropriate number of MoniKas with sufficient MamBo-experience (*n* = 3) was available. After a delay in setting up the MamBo-structures, the first interviews with the DM and CM were conducted from May until July 2019. Two management consultants were invited to an interview once in summer 2019.

All interviews will be recorded and transcribed verbatim and pseudonymised [11]. The data will be analysed using qualitative content analysis according to Mayring [12, 13]. For this, the software MAXQDA will be used.

### Quantitative evaluation

#### Quantitative evaluation design

In order to measure effectiveness and treatment effects, postal survey data of all physicians, practice staff as well as patients enrolled in MamBo, will be conducted as a longitudinal study with two time-points. The first questionnaire (t0) is surveyed immediately after the enrolment into the project. The second questionnaire (t1) was originally surveyed 1 year later. As we had difficulties in reaching the required number of patients, the funder proposed to change the intervention period from 1 year to 6 months in the course of the extension application. Due to the fact that the majority of the MoniKa intervention takes place within the first 6 months after enrolment in MamBo and that the MamBo structure is more established in 2020 than in previous years, the evaluation team assumes that a comparison of the groups is still possible and agreed to shorten the period of patient examination. Patients enrolled after November 2019 will, therefore, receive the t1 after 6 months.

Depending on the doctors’ assessment of the patients’ health condition and needs as well as the patients’ wishes, the included patients receive either a MoniKa-home visit, a short MoniKa-call or no MoniKa contact at all. The latter do not receive an additional new form of care and will be considered as the non-treatment group. However, it is expected that they profit indirectly from the MamBo structures on the organisational level. Thus, the patients’ survey can be defined as a cohort study with follow-up and a non-randomized treatment. Non-participating physicians will also be surveyed cross-sectional.

To evaluate the cost-efficiency and effectiveness of the care model secondary claim data will be used. For this, a quasi-experimental cohort study will be conducted. Multimorbid patients (control group) will be compared with multimorbid MamBo-patients (intervention group).

In addition, documentations of the physician network with records of the process-data of the CM provides information about the implementation process.

### Questionnaire data

#### Sampling

Participants will be approached through the data trustee of the evaluation team, who is the only one with access to the physicians’ practice and patients’ contact addresses for the surveys. With their enrolment into the MamBo-care model, each participating physician, practice staff and patient receive a written letter of consent to participate in the first and second surveys. Addresses of physicians who are members of the physicians’ network but do not participate in MamBo will be provided by the CM to the trustee. The written declaration of consent and the questionnaire will be sent out in parallel. In order to guarantee high quality, standards for the questionnaire development [14–16] methods of pretesting [17] and the Dillman’s Total Design Method for achieving a possibly high response rate [18] were used.

Since the given consent of the physicians’ survey in the first study year was approx. 50% and is a lot lower in the practice staff survey, a response from approx. 20 and 40 participants are expected for t0. The first questionnaire of all participating physicians and their practice staff started in the first half of 2018. The non-participating physicians survey was conducted in summer 2019 (*n* ~ 60).

The t0 questionnaire of patients shows a very high consent rate and a response rate of approx. 80%. With the high response rate and incentives provided, low panel attrition is expected (approx. 20%). As an incentive, a postage stamp is enclosed with the t1 questionnaire. The survey of patients also started in the first half of 2018. The last enrolment for the t0 questionnaire will be in April 2020 (n ~ 1900), accordingly approx. 1500 patients will be surveyed at both, t0 and t1.

#### Data collection and analysis

To determine the perceived benefits of the care model the physician survey includes expectations of the care model (t0) or rather the fulfilment of expectations (t1). Besides that, practice characteristics (e.g. number of MFAs, joint practice/single practice) and process data (e.g. workload, information procurement) are collected. In the survey of non-participating physicians, reasons for non-participation are collected additionally. By distinguishing between participating and non-participating physicians, but also between “early adopters” and “late adopters”, it can be determined whether and if so which characteristics are significantly different between these groups. With the second survey, a pre-post comparison is possible to examine which factors have changed as a result of participation.

With the first patient survey (t0), sociodemographic characteristics, health-related characteristics (e.g. state of health, mobility, well-being), general characteristics (e.g. social support, coping, living environment), satisfaction with previous care, reasons for participating in MamBo and expectations are recorded. Translated and validated standard scales used in the physicians and patients surveys are listed in Table 2.

**Table 2:** Standardized scales used in the postal surveys

Scale	Outcome
**Physicians survey**	
HSOPS_M Hospital Survey on Patient Safety Culture for Hospital Management - overall perceptions of safety) [19]	patient safety
Dispositional Resistance to Change Scale - routine seeking [20]	openness to innovations
The workload in Nursing scale captures - psychophysical overload [21]	psychophysical burden of workload
**Patients survey**	
WHO 5-items Well-being Scale [22]	wellbeing
PACIC - Patient Assessment of Chronic Illness Care shortened form [23]	patients’ perceptions of the quality of care they have received for their chronic conditions
EFK-HPC Questionnaire on Disease Processing - Acting, problem-oriented Coping [24]	current coping efforts
Patient questionnaire of cologne (subscale) [25]	burden of disease
HL-COM -Health Literacy sensitive Communication [26]	health literacy sensitive communication
Medication Adherence Rating Scale (MARS) [27]	drug compliance
BS6 - Brief Social Support Scale (BS6) [28]	social support
PHQ-2-Patient Health Questionnaire 2 [29]	depression
EORTC-QLQ-C30 (subscales) [30]	quality of life regarding physical function and global health

In the second survey (t1) only the time-variant measures remain. In addition, questions regarding life events and the MamBo-intervention are added. Primary outcomes are patients’ perceptions of the quality of care, social support, drug compliance and wellbeing. The analysis of the survey at t0 examines whether and in which characteristics participating patients differ from each other at their point of enrolment. With the second survey, a pre-post comparison within one group and a pre-post comparison between the treatment groups and the non-treatment group will be possible.

For data preparation, Tele-form® software has been used. The quantitative data will be analysed using regression-based methods. Structural Equation Modeling (SEM) is used to study intermediate social factors. Difference-In-Differences (DiD) estimation are used to inspect the effect of the MoniKa intervention on e.g. patient satisfaction. Confounder adjustment will be achieved by using inverse-probability weights (IPW), to adjust for the probability of being visited by a MoniKa (treatment group). Various covariates will be specified as confounders like patients’ morbidity characteristics (patients’ mobility, age and years of being chronically ill). In addition, the patients’ trust in the physicians will also be accounted for. To assess comparability, a subgroup analysis will be performed between patients who completed the t1 questionnaire after 1 year and patients who received it after 6 months. Data will be analysed using StataMP 15.1.

### SHI-claim data

#### Sampling

Claim data will be used to assess and compare the costs and use of health services between MamBo patients and patients in standard care. For this purpose, the SHI “PronovaBKK” provides anonymised claim data of the MamBo-population. A statistical twin with data available to the German Health Risk Institute (HRI) [31] is determined for each MamBo participant using the propensity score matching (PSM) method [32]. Matching is performed by the HRI according to criteria such as age, gender and diagnosis.

A sample size of *n* = 2617 corresponding to a statistical power of 79.2% was aimed, given an unpaired t-test, a Cohen’s d of 0.068 and a significance level of 0.05. However, the patients’ enrolment did not correspond to the expected sample size goals, which caused reduced statistical power. To ensure maximum statistical power, the evaluation design of the SHI-claim data analysis has been adopted to the different temporal availabilities of the key parameters. One part can be delivered within 3 months. The other part is available with a delay of up to 9 months. Therefore, group 1 (*n* = 1.975) was closed on 01.04.2019 and group 2 (*n* = 2365) was closed on 01.10.2019. By this, sufficiently high power is guaranteed. The power for Group 1 is about 68% and for Group 2 about 76%.

#### Data collection and analysis

Both patient-related (e.g. improvements of care,) as well as payer-related (e.g. hospitalizations, utilization of outpatient care) objectives will be considered in the analysis. With MamBo a mean cost reduction of 12,5% is expected due to reduced hospitalization rates. For all variables, a comparison is made between the intervention group and the twin group with significance tests. The selection of the appropriate statistical test depends, among others, on the type (binary/continuous) and distribution of the respective variables.

### RGL process data (physician’s network)

The number of participating physicians and of patients they enroll, all processes of the CM including activities of the MoniKa and the number and contents of the continuous improvement process meetings are documented in the RGL. This data is handed in quarterly to the evaluating institute in aggregated form. This data enables the evaluation of the success of the implementation determined by successful MoniKa delegation, quality and quantity of MoniKa visitations and sustainable physicians’ participation.

## Discussion

The “MamBo” care model offers a new form of coordinated, managed and cross-sectoral care for multimorbid patients. There are already coordinated programmes such as the DMPs in Germany [7]. While DMPs only promote isolated diseases, MamBo addresses the needs of multimorbid patients. This study evaluates a new form of care formatively and summatively. By conducting a multi-perspective study with several data sources and study results, patient-, provider- and cost-unit-related goals are addressed. In addition, confounding factors are taken into account by considering various additional data sources, such as process data and SHI claim data. As far as we know, the study will also be the first to formatively evaluate the benefits of a management consultancy that supports the change management as an implementation strategy for complex health innovations.

However, it should be noted that this study is a health services research study. Thus, this is an evaluation study that primarily investigates the effectiveness of a new care model. Consequently, the study shows its limitations. There will be an increased risk of selection bias due to the quasi-experimental study design in which participating physicians enroll patients and only selected patients receive MoniKa treatment. Additionally, within the formative evaluation just willing and thus mostly active physicians can be interviewed. Due to difficulties in the recruitment process, the evaluation-design had to be adopted during the project period. The occurrence of group differences between patients who completed the t1 survey after one year and patients who were interviewed after a six-month intervention cannot be completely excluded.

## Data Availability

It is planned to submit the results of the formative and summative evaluation for publication in peer-reviewed journals and to present them at national and international conferences. The dissemination will also be supported by professional public relations activities. The anonymous datasets generated during the current study may be made available from the corresponding author on a reasonable request. Protocol modifications will be communicated to relevant parties.

## Abbreviations

CM: Care Management
CIP: Continuous Improvement Process
DM: Demand Management
DiD: Difference-In-Differences
DMP: Disease Management Programme
GP: General Practitionairs
HRI: German Health Risk Institute
IPW: Inverse-Probability Weights
MoniKa: Monitoring and Coordinating Assistant
MamBo: People with Multimorbidity in Outpatient Care: Patient-Focused and Needs-Oriented Healthcare Management”
PSM: Propensity Score Matching
RGL: Regional Health Network
SHI: Statutory Health Insurance
SEM: Structural Equation Modeling
